# Phosphorylation of Pnut in the Early Stages of *Drosophila* Embryo Development Affects Association of the Septin Complex with the Membrane and Is Important for Viability

**DOI:** 10.1534/g3.117.300186

**Published:** 2017-10-17

**Authors:** Katarina Akhmetova, Maxim Balasov, Anton Svitin, Elena Chesnokova, Matthew Renfrow, Igor Chesnokov

**Affiliations:** * Department of Biochemistry and Molecular Genetics, School of Medicine, University of Alabama at Birmingham, Alabama 35294; † The Federal Research Center Institute of Cytology and Genetics, Novosibirsk 630090, Russian Federation; ‡ Novosibirsk State University, 630090, Russian Federation

**Keywords:** *Drosophila*, septins, Pnut, phosphorylation, Orc6

## Abstract

Septin proteins are polymerizing GTPases that are found in most eukaryotic species. Septins are important for cytokinesis and participate in many processes involving spatial modifications of the cell cortex. In *Drosophila*, septin proteins Pnut, Sep1, and Sep2 form a hexameric septin complex. Here, we found that septin protein Pnut is phosphorylated during the first 2 hr of *Drosophila* embryo development. To study the effect of Pnut phosphorylation in a live organism, we created a new *Drosophila pnut* null mutant that allows for the analysis of Pnut mutations during embryogenesis. To understand the functional significance of Pnut phosphorylation, *Drosophila* strains carrying nonphosphorylatable and phospho-mimetic mutant *pnut* transgenes were established. The expression of the nonphosphorylatable Pnut protein resulted in semilethality and abnormal protein localization, whereas the expression of the phospho-mimetic mutant form of Pnut disrupted the assembly of a functional septin complex and septin filament formation *in vitro*. Overall, our findings indicate that the controlled phosphorylation of Pnut plays an important role in regulating septin complex functions during organism development.

Septins belong to a family of polymerizing GTP binding proteins that are essential for cytokinesis in many organisms and are recognized as important components of the cytoskeleton. They localize primarily to the cell membranes and participate in many cellular processes, including cell division, movement and polarity, spindle alignment, ciliogenesis, secretion, cell–pathogen interaction, and cytoskeletal dynamics (Glotzer 2001; Spiliotis and Nelson 2006; Weirich *et al.* 2008; Hu *et al.* 2010; Kim *et al.* 2010; Estey *et al.* 2011; Saarikangas and Barral 2011; Mostowy and Cossart 2012; Bridges and Gladfelter 2015). The number of septin genes varies greatly in different organisms; however, the basic septin functions are similar between the species. Often, septins form scaffolds to recruit other proteins, or membrane diffusion barriers, thereby compartmentalizing discrete cellular domains (Estey *et al.* 2011; Saarikangas and Barral 2011; Hall and Russell 2012; Mostowy and Cossart 2012; Caudron and Barral 2009; Versele and Thorner 2005; Chao *et al.* 2014; Clay *et al.* 2014; Khan *et al.* 2015; Trimble and Grinstein 2015). Considerable diversity of biological processes served by septins is based on the ability of these proteins to form complexes and filaments.

*Drosophila melanogaster* has five septins: Sep1, Sep2, Pnut, Sep4, and Sep5. Three of them, Pnut, Sep2, and Sep1 (homologs of human SEPT7, SEPT6, and SEPT2, respectively), form a heteromeric six-subunit complex consisting of two of each septin subunits (Field *et al.* 1996; Huijbregts *et al.* 2009). By interacting end-to-end, septin complexes form nonpolar filaments that can further assemble into higher order structures, such as rings. Filament assembly and breakdown are dynamic processes that are regulated by many factors, such as GTP binding and hydrolysis (Sirajuddin *et al.* 2009; Bertin *et al.* 2008; Mendoza *et al.* 2002; Vrabioiu *et al.* 2004), interacting partners (Joberty *et al.* 2001; Dekker *et al.* 2008; Huijbregts *et al.* 2009; Sadian *et al.* 2013; Akhmetova *et al.* 2015), and post-translational modifications (Hernandez-Rodriguez and Momany 2012); however, the details and mechanisms are not completely understood.

Multiple post-translational modifications, including SUMOylation, acetylation, and phosphorylation, have been reported for septins (Hernandez-Rodriguez and Momany 2012). The most common modification is phosphorylation: numerous septins are phosphorylated at various times during the cell cycle, which can trigger septin rearrangement or disassembly (Hernandez-Rodriguez and Momany 2012). For instance, in *Saccharomyces cerevisiae*, Cdc3, Cdc11, Cdc10, and Shs1 can all be phosphorylated (Dobbelaere *et al.* 2003; Ficarro *et al.* 2002; Mortensen *et al.* 2002; Tang and Reed 2002; Versele and Thorner 2004; Chi *et al.* 2007). Mutations in phosphorylated residues (mostly serines) result in budding defects and impaired septin higher order structures (Tang and Reed 2002; Versele and Thorner 2004; Garcia *et al.* 2011). In *Candida albicans* and filamentous fungus *Ashbya gossypii*, mutations in phosphorylated residues affect septin ring dynamics and result in altered hyphal and spore morphology (Sinha *et al.* 2007; Meseroll *et al.* 2012, 2013). Little is known about the phosphorylation of septins in higher eukaryotes compared to fungi. Mammalian SEPT8, SEPT4, SEPT5, SEPT9, SEPT2, and SEPT3 are found to be phosphorylated in different tissues (Estey *et al.* 2013; Koch *et al.* 2015; Amin *et al.* 2008; Sitz *et al.* 2008; Shiryaev *et al.* 2012; Xue *et al.* 2004; Taniguchi *et al.* 2007; Yu *et al.* 2009; Scholz *et al.* 2015); however, in many cases, the functional significance of these modifications remains unclear.

In a present work, we found that *Drosophila* septin Pnut (homolog of human SEPT7) is phosphorylated during the first 2 hr of embryogenesis. To study the functional and biological effects of Pnut phosphorylation in *Drosophila*, we created a new *pnut* null mutant. Fly strains carrying transgenes with nonphosphorylatable or phospho-mimetic *pnut* mutations were established. Our studies suggest that regulated Pnut protein phosphorylation is important for *Drosophila* embryogenesis and viability and has a strong effect on septin localization and function.

## Materials and Methods

### Generation of a new pnut null mutant

Our null mutation of the *pnut* gene was created by the excision of a *P* element-based transposon *P*{*SUPor-P*}*pnut^KG00478^* (FBti0023313). This transposon is mapped 67 bp downstream of the *pnut* start codon. To initiate excision, males *y^1^*;*P*{*y^+mDint2^w^BR.E.BR^ = SUPor-P*}*pnut^KG00478^/SM6a* (Bloomington stock 14354) were crossed to females of the “jump” stock *y^1^w^1118^*;*CyO*, *PBac*{*w*+^mC^*=Delta 2-3. Exel*}*2*/*amos^Tft^*, bearing Δ2-3 transposase on a second chromosome, marked by *Curly*. F1 *Curly* progeny *y^1^w^1118^*;*P*{*y^+mDint2^ w^BR.E.BR^ = SUPor-P*}*pnut^KG00478^*/*CyO*, *PBac*{*w+mC*=*Delta 2-3. Exel*}*2* were collected and crossed to *y^1^w^1118^*; *If*/*CyO* females. The resulting F2 progeny was screened for white-eyed flies. White-eyed flies were crossed individually to *y^1^w^1118^*; *If*/*CyO* to set up stocks *pnut^mut^*/*CyO*. The genomic DNA of these mutants was isolated. Mutations were confirmed by sequence determination following PCR amplification with *pnut* primers: forward 5′-ACTAGTAGGAGTCGGGCTAATAAC-3′ and reverse 5′-CCCGGATCCTTAGAACAGACCCTTC-3′.

### Cloning

Pnut mutations that carried a replacement of T509 and S517 with alanine residues to prevent phosphorylation (Pnut-T509A/S517A), or with glutamate residues to mimic constitutive phosphorylation (Pnut-T509E/S517E), were generated following the Stratagene site-directed mutagenesis protocol (https://www.agilent.com/cs/library/usermanuals/public/200523.pdf). The plasmids for *Drosophila* transformation were generated as follows. The DNA fragments containing the 1.7 kb 5′-UTR of the *pnut* gene (potential native promoter) followed by *FLAG-pnut* wild-type or mutated cDNA were each inserted into *pCasper3* with deleted *UAS* sequences. The same constructs were used to obtain stable S2 cell lines. Recombinant baculoviruses were generated as described (Huijbregts *et al.* 2009; Akhmetova *et al.* 2015). For electron microscopy (EM) experiments, *orc6* wild-type cDNA was inserted into the *pQE30* expression vector as described earlier (Akhmetova *et al.* 2015). All constructs were confirmed by DNA sequencing.

### Transgenic animals and rescue experiments

All crosses were carried out at 25° under standard conditions except where indicated. *Canton S* fly stock was used as a wild-type control. *pCasper*-based vectors under control of the native *pnut* promoter containing wild-type (*FLAG-pnut-WT*) and mutated (*FLAG-pnut-T509A/S517A* and *FLAG-pnut-T509E/S517E*) *pnut* transgenes were injected into *w^1118^ Drosophila* embryos. The expression of tagged proteins was verified by immunoblot analysis with anti-FLAG antibody (Supplemental Material, Figure S1 in File S1). Homozygous fly stocks *w^1118^*;*pnut^mut1^*/*pnut^mut1^*;*FLAG-pnut-WT*, *w^1118^*;*pnut^mut1^*/*pnut^mut1^*;*FLAG-pnut-T509A/S517A* and *w^1118^*;*pnut^mut1^*/*pnut^mut1^*;*FLAG-pnut-T509E/S517E* were set up. Several independent transgenic lines were established for each transgene, and all gave similar phenotypes. One strain corresponding to each transgene was used for subsequent experiments. For the pupae–imago viability assay, females were allowed to lay eggs overnight. The next day, the flies were removed and the number of laid eggs in each vial was counted. Then, the percentages of formed pupae and enclosed imagos were calculated. For embryonic viability assay, 0–4 hr eggs were collected on molasses plates and counted. After 48 hr, the percentages of hatched larvae were calculated. In both experiments, the Student’s *t*-test was used to determine *P*-values.

### Immunofluorescent analysis of Drosophila embryos

Females were allowed to lay eggs on molasses plates for 2 hr. Embryos were collected from plates, rinsed 2× in wash solution (0.7% NaCl and 0.3% Triton X-100), 2× with water, dechorionized in 50% bleach for 4 min, and then rinsed twice each with wash solution and water. Water was removed and fixative was added (1:1 ratio of 4% paraformaldehyde in PBS:n-Heptane). Embryos were shaken vigorously for 1 min and placed on a rotator for 25 min. For standard devitellinization, the lower phase (fixative) was removed, substituted with the same amount of methanol, and vigorously shaken for 1 min to remove the vitelline membrane. The upper liquid (heptane) was removed, embryos were washed 3× with methanol, and stored at −20°. For manual devitellinization (without methanol), after formaldehyde fixation, embryos were transferred to a small piece of Whatman paper, dried for 30 sec, transferred to double-stick tape on the bottom of a small petri dish, and immediately covered with PBST solution (phosphate buffer with 0.2% Triton X-100). The vitelline membrane was removed using thin dissecting needles. After devitellinization (using either of methods), embryos were washed with PBST and blocked with PBST supplemented with 10% goat serum for 1 hr. The embryos were incubated with primary antibody overnight at 4°, washed 3× for 15 min with PBST, incubated with secondary antibody for 2 hr at 30°, washed 3× for 15 min, counterstained with DAPI (4ʹ,6-diamidino-2-phenylindole, Roche), washed with PBS, and mounted in Fluoromount-G (SouthernBiotech). Primary antibodies used: anti-Pnut rabbit (Huijbregts *et al.* 2009) (1:400), anti-Pnut mouse (4C9H4: G.M. Rubin, DSHB, 1:60), anti-FLAG mouse (clone M2, F1804, 1:300; Sigma, St. Louis, MO), and anti-Sep1 rabbit (Akhmetova *et al.* 2015) (1:300). Secondary antibodies conjugated to Alexa488 or Alexa568 (1:300; Molecular Probes, Eugene, OR) were used. Alexa Fluor 568 phalloidin (Molecular Probes, 1:40) was used to visualize actin. The images were taken with an Olympus BX61 motorized upright microscope fitted with a BX-DSU disc scan unit.

### Drosophila S2 tissue culture cells

The *Drosophila* S2 cells were cultured at 27° in Shields and Sang M3 medium (Sigma) supplemented with 5% fetal bovine serum. To arrest cells in mitosis, S2 cells were incubated with 30 μM colchicine (Sigma) for 14–24 hr. To estimate the enrichment of metaphase stage, cells were stained with DAPI as described above. For immunoblot analysis with antibody against Pnut (Huijbregts *et al.* 2009), S2 cells were collected, centrifuged, and resuspended in a lysis buffer [50 mM HEPES, pH 7.6, 2.5 mM MgCl_2_, 150 mM NaCl, 1 mM EDTA, 0.02% Triton X-100, 1 × Halt protease and phosphatase inhibitor cocktail (Thermo Scientific)].

### Generation of stable Drosophila cell lines

To obtain stable cell lines expressing FLAG-tagged Pnut, 3 × 10^6^ S2 cells were seeded in a six-well dish. Next, 2 μg of the *pCasper*-based plasmid carrying wild-type or a mutant form of FLAG-tagged Pnut were cotransfected with 0.2 μg of the plasmid *pCoHygro* using a Cellfectin reagent (Invitrogen, Carlsbad, CA) or Insect Genejuice (Novagen). Sixteen hours post-transfection, cells were divided over ten 100 mm dishes and allowed to settle overnight. From this point on, cells were cultured in a media containing 50 μg/ml Hygromycin B. After 3–4 wk, the colonies were isolated, expanded, and analyzed for protein expression by immunoblotting and immunofluorescent microscopy with anti-FLAG antibody.

### RNAi in Drosophila S2 cells

Double stranded RNA (dsRNA) was obtained by using the Megascript kit (Ambion). Primers complementary to the 3′-UTR of the *pnut* transcript (5′-CGGCCAGT GAATTGTTTAATACGACTCACTATAGGGACGCTCAAAACCCCCATTCCC-3′ and 5′-CGGCCAGTGAATTGTTTAATACGACTCACTATAGGGTCGCCTCGCACTCGTACATTC-3′) flanked with the T7 promoter were used. Next, 1 × 10^6^ S2 cells seeded on a cover slip in a well of a six well- dish were inoculated with 15 μg of dsRNA in 1 ml serum-free M3 medium. After a 1 hr incubation, 1 ml of medium supplemented with 10% fetal bovine serum was added to the culture. After 120 hr, cells were fixed with 2% formaldehyde in PBS. Then, the cells were stained for FLAG (clone M2, F1804; Sigma) and counterstained with DAPI (Roche). Cover slips were mounted with Fluoromount-G (SouthernBiotech) and analyzed by fluorescence microscopy. The RNAi efficiency was tested by immunoblotting with antibody against Pnut (Huijbregts *et al.* 2009).

### Immunoprecipitation (IP) experiments

Flies of genotype *pnut^mut1^/pnut^mut1^*;*FLAG-pnut* wild-type or with mutated phosphorylation sites were placed on molasses plates and allowed to lay eggs for 2 hr. 50 embryos were collected from each plate, manually dechorionized, and ground in 50 μl of IP buffer [50 mM HEPES, pH 7.6, 2.5 mM MgCl_2_, 150 mM NaCl, 1 mM EDTA, 0.02% Triton X-100, 1 × Halt protease and phosphatase inhibitor cocktail (Thermo Scientific)] using a glass micro grinder (catalog number 357848; Wheaton). The lysates were clarified by spinning for 5 min at 10,000 × *g*, and 150 μl of IP buffer with 2 μl of anti-FLAG antibodies (Sigma, clone M2, catalog number F1804) were added to extracts. After 4 hr of incubation at room temperature (22°), 10 μl of Protein G Sepharose (Amersham Biosciences, Piscataway, NJ) were added and reactions were incubated for 1 hr. Beads were then washed three times with IP buffer and boiled in a loading buffer. Immunoprecipitated material was analyzed by SDS-PAGE followed by western blotting using antibodies against Pnut, Sep1, and Sep2 (Huijbregts *et al.* 2009).

### In vitro assembly of a septin complex

Recombinant baculoviruses were generated and septin complexes were reconstituted as described (Huijbregts *et al.* 2009; Akhmetova *et al.* 2015). Purified complexes were stored in a buffer containing ∼500 mM KCl. Next, 2 μg of septin complexes containing wild-type or mutated Pnut protein were diluted in 200 μl of pull-down buffer (25 mM HEPES, pH 7.6, 2 mM MgCl_2_, and 10 mM imidazole, with the addition of 50 mM or 300 mM KCl). Following this, 10 μl of HisPur Ni-NTA Resin (Thermo Scientific) were added and the reactions were incubated for 1 hr at room temperature (22°). Beads were washed three times with a pull-down buffer, after which bound proteins were eluted with a pull-down buffer containing 250 mM imidazole and analyzed by a silver-stained SDS-PAGE.

### EM

Preparations of recombinant septin complexes containing wild-type or mutated Pnut were diluted to 40 ng/μl in GTPase buffer (25 mM HEPES-KOH, pH 7.6, 2 mM MgCl_2_, and 1 mM EGTA) with 50 mM or 300 mM KCl. Next, 40 ng/μl wild-type Orc6 (the Origin Recognition Complex subunit 6) and 400 μM GTP were added to the reaction where indicated. Purification of wild-type Orc6 protein has been described (Akhmetova *et al.* 2015). The samples were incubated at 22° for 2 hr, applied to copper Formvar carbon-coated grids, and incubated for 3 min. Next, the excess solution was removed and proteins were fixed with 1% uranyl acetate in water for 1 min. Then, the fixative was removed and grids dried. The images were taken on a FEI Tecnai F20 electron microscope operated at 200 kV.

### Embryonic extract preparation

Population cages were set up for wild-type flies. Before egg collection, flies were fed with yeast paste on molasses plates for 2 d to stimulate egg laying. On day three, fresh molasses plates were placed in cages and flies were allowed to lay eggs for 15 min. Embryos were then aged for the time indicated, collected, washed with wash solution (0.7% NaCl and 0.3% Triton X-100), dechorionized with 50% bleach, and ground in a lysis buffer [50 mM HEPES, pH 7.6, 2.5 mM MgCl_2_, 100 mM KCl, 1 mM EDTA, 10% glycerol, 1 × Halt protease and phosphatase inhibitor cocktail (Thermo Scientific)]. Lysates were clarified by spinning for 5 min at 10,000 × *g* and analyzed by immunoblots using an antibody against Pnut (Huijbregts *et al.* 2009).

### Mass spectrometry identification of Pnut phosphorylation sites

Embryo lysates were immunoprecipitated with anti-Pnut antibody and precipitates were then separated by SDS-PAGE. Individual protein bands (upper and lower) were excised from the gel with a razor blade. Gel bands were subsequently reduced with 10 mM dithiothreitol for 45 min at 37°, alkylated with 50 mM iodoacetamide for 45 min at 37°, and digested overnight with both trypsin (Pierce, Rockford, IL) and AspN (Sigma) at 37°. Peptides were extracted from the gel using 50% acetonitrile and concentrated in a speedvac. The recovered peptides were loaded onto a 100 μm diameter, 10.5 cm pulled tip packed column with Jupiter 5 μm C18 reversed-phase beads (Phenomenex) using a Micro AS autosampler and LC nanopump (Eksigent). Parallel runs for upper and lower band digests were analyzed via a linear ion trap-Fourier transform ion cyclotron resonance hybrid mass spectrometer (LTQ FT, Thermo Scientific) using either CID or ECD fragmentation. A gradient of H20-acetonitrile in 0.1% formic acid was run from 5 to 40% over the course of 50 min at 650 nl min^-1^ (% H20/acetonitrile). LTQ FT CID parameters were set as previously described (Renfrow *et al.* 2007). ECD parameters were set with the Xcalibur software under the following arbitrary parameters and millisecond durations: 100 ms ECD at power level 2.25 and a 90 ms delay before IRMPD stimulation for 60 ms at power level 15. *Drosophila* peptides from both the tryptic and AspN digests were initially identified with SEQUEST (Eng *et al.* 1994), MASCOT (Perkins *et al.* 1999), and Protein Prospector (Clauser *et al.* 1999) software prior to manual inspection of modified and unmodified peptides. Pnut was unambiguously identified within its respective excised gel bands with >20 unique peptides.

### Data availability

The authors state that all data necessary for confirming the conclusions presented in the article are fully represented within the article. The strains and plasmids are available upon request.

## Results

### Pnut is phosphorylated during early embryogenesis

**Figure 1:**
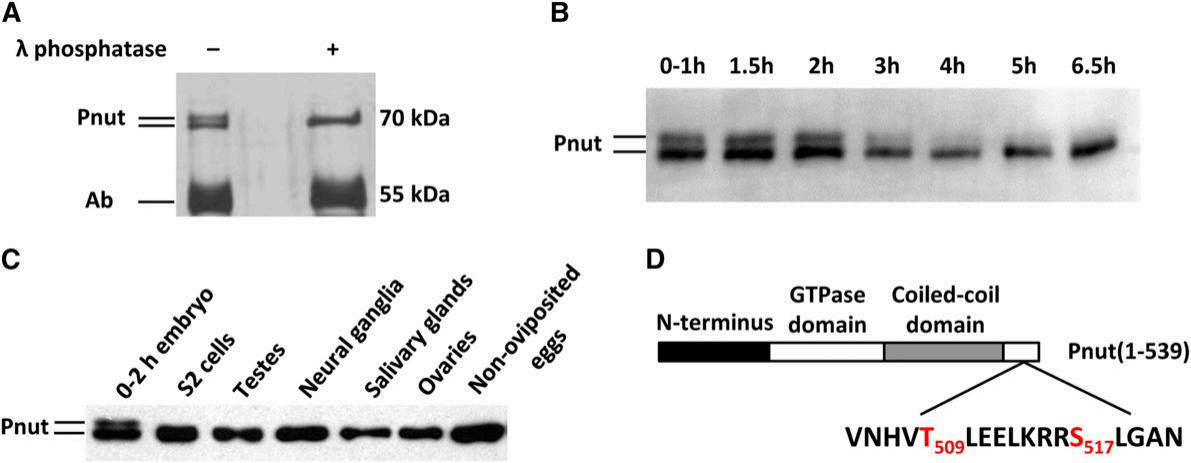
Pnut is phosphorylated during early *Drosophila* embryogenesis. (A) Pnut protein was immunoprecipitated from the 0–2 hr embryonic extract using anti-Pnut antibody. Immunoprecipitated material was treated with λ phosphatase where indicated and analyzed by silver-stained SDS-PAGE. (B) Extracts from embryos at different time points of development were analyzed by immunoblot with anti-Pnut antibodies. (C) Extracts from different organs and tissues, as well as S2 tissue culture cells, were subjected to immunoblot using antibody against Pnut. (D) Schematic representation of Pnut protein structure. Phosphorylated *in vivo* residues (shown in red) and their relative locations are shown. Ab, antibody; SDS-PAGE, sodium dodecyl sulfate-polyacrylamide gel electrophoresis.

We found a potential post-translational modification of the Pnut protein during large-scale isolation of the septin complex from developing *Drosophila* embryos. We observed that Pnut protein in purified septin fractions often migrated as a double band during separation in SDS-PAGE silver-stained gels. To investigate this phenomenon in more detail, we prepared extracts from embryos at different stages of development. The septin complex was immunoprecipitated from the extracts prepared from embryos collected at the early (0–2 hr) stages of development using the antibody raised against the *Drosophila* Pnut protein (Figure 1A). Pnut protein from the pull-down material migrated as a double band (Figure 1A, first lane). The treatment with λ phosphatase (Figure 1A, last lane) resulted in a shift of mobility from the upper to the lower band, suggesting that this modification was due to the phosphorylation of Pnut protein.

The first 13 rounds of nuclear division in *Drosophila* embryos occur in the absence of cytokinesis. The first nine divisions (0–1.5 hr of development) take place in the embryo interior, and the only membrane that exists at this stage is the outer membrane of the embryo itself. During the syncytial blastoderm stage, which lasts for ∼30 min (1.5–2 hr of embryo development), nuclei migrate to the egg periphery and undergo four mitotic cycles. At this stage, all the nuclei are tightly packed in a shared cytoplasm. To avoid spindle collisions during mitoses, plasma membrane ingressions called pseudocleavage furrows form transiently between nuclei. It is only during the interphase of 14th mitotic cycle that each cell becomes surrounded by a membrane. This process is called cellularization and lasts for ∼1 hr (2–3 hr of embryo development) (Foe 1989).

To examine the dynamics of Pnut phosphorylation during embryogenesis, we prepared total extracts from fly embryos at different time points of development and analyzed them by immunoblotting using the antibodies against Pnut (Figure 1B). The intensity of the upper band was most pronounced (up to 40% of total Pnut) during the first 2 hr after the eggs were laid. This band diminishes significantly after 2 hr of development. According to the timing of early *Drosophila* embryo development, the phosphorylated form of Pnut appears from the very beginning of embryogenesis, persists up to the cellularization stage, and greatly decreases after the end of cellularization, when individual cells surrounded by membranes are finally formed.

To address the question of whether the phosphorylated form of Pnut may be maternally deposited, we analyzed extracts isolated from fully matured nonoviposited eggs (dissected from ovaries). No extra bands corresponding to the phosphorylated form of Pnut were observed at this stage (Figure 1C, last lane), suggesting that Pnut phosphorylation occurs during the first hour of embryogenesis. Pnut phosphorylation was not detected in *Drosophila* culture cells or in the extracts isolated from different *Drosophila* organs and tissues (Figure 1C).

The putative phosphorylated form of Pnut was isolated and subjected to mass spectrometry analysis. We found that the serine residue at a position 517 and the threonine at position 509 were phosphorylated. The protein sequence surrounding these phosphorylated residues is as follows: VNHV**T_509_**LEELKRR**S_517_**LGAN (Figure 1D). This motif is positioned at the very end of the C-terminus of the Pnut protein, just outside the predicted coiled-coil domain.

### Generation of pnut null mutant in Drosophila

The lethal *pnut* null allele, *pnut^XP^* (Neufeld and Rubin 1994), available at the Bloomington *Drosophila* Stock Center, represents a deletion of at least 17 kb that includes the *pnut* gene. Homozygotes for *pnut^XP^* can be rescued to the imago stage by the expression of a wild-type *pnut* transgene (Neufeld and Rubin 1994; Adam *et al.* 2000; Akhmetova *et al.* 2015). However, rescued females are sterile, most probably due to the large size of the deletion affecting nearby genomic regions. Female sterility does not allow for the studying of the effect of different *pnut* mutations on embryogenesis and early development.

**Figure 2:**
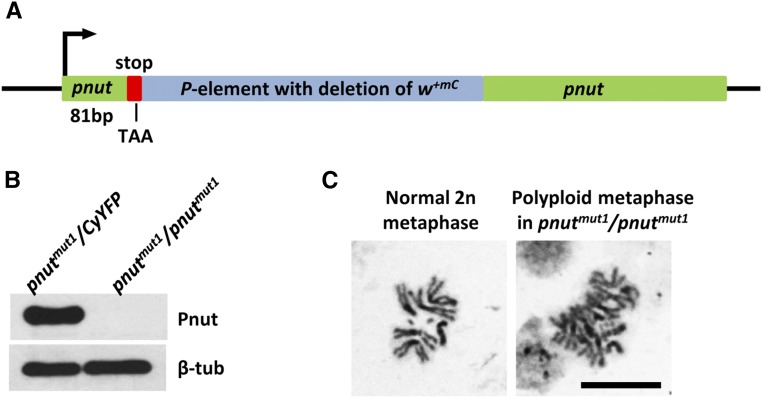
Characterization of a new null *pnut^mut1^* mutation. (A) Partial excision of a *P* element resulted in formation of a TAA stop codon at position 82 upstream of the *pnut* start codon. (B) Immunoblotting analysis using antibody against Pnut shows the absence of *pnut* product in larval tissues of *pnut^mut1^* homozygotes as compared to heterozygotes. Antibodies against β-tubulin (β-tub) were used as a loading control. (C) Neural ganglia of *pnut^mut1^* homozygotes contain polyploid cells similar to *pnut^XP^* deletion. Bar, 10 µm.

To overcome this limitation, we used the method of *P* element imprecise excision to create new *pnut* mutations. A *P* element in *y^1^*;*P*{*SUPor-P*}*pnut^KG00478^/SM6a* was excised by Δ2-3 transposase. One of the obtained excisions, *pnut^mut1^*, contained a partial deletion of the *P* element and resulted in the formation of a stop codon 82 bp downstream of the *pnut* start codon (Figure 2A). This excision did not extend into the *pnut* gene but resulted in the loss of a *mini-white* marker of the *P* element, which allowed us to detect the deletion by eye color. The absence of a full-size Pnut was verified by immunoblotting (Figure 2B). This new *pnut^mut1^* allele is third instar lethal. It is not complemented by the *pnut^XP^* null allele and has a phenotype similar to that of the *pnut^XP^* mutation, including polyploidy (Figure 2C) and the absence of imaginal discs (larval epithelial structures that differentiate into imago organs). Importantly, *pnut^mut1^* homozygous female flies can be rescued by the expression of the wild-type *pnut* transgene. These females with the genotype *pnut^mut1^/pnut^mut1^*;*pnut-WT*, unlike rescued *pnut^XP^* mutants, are fertile, allowing the analysis of various *pnut* mutations during embryogenesis.

### The analysis of the effect of Pnut phosphorylation on Drosophila survival

To study the effects of Pnut phosphorylation *in vivo*, we generated nonphosphorylatable and phospho-mimetic Pnut mutations. Two transgenes were created using site-directed mutagenesis. To make a nonphosphorylatable mutant, we substituted phosphorylation substrates T509 and S517 with alanine residues resulting in a *pnut-T509A/S517A* transgene. For the phospho-mimetic mutation, the target amino acids were mutated to glutamic acids, resulting in *pnut-T509E/S517E*.

**Figure 3:**
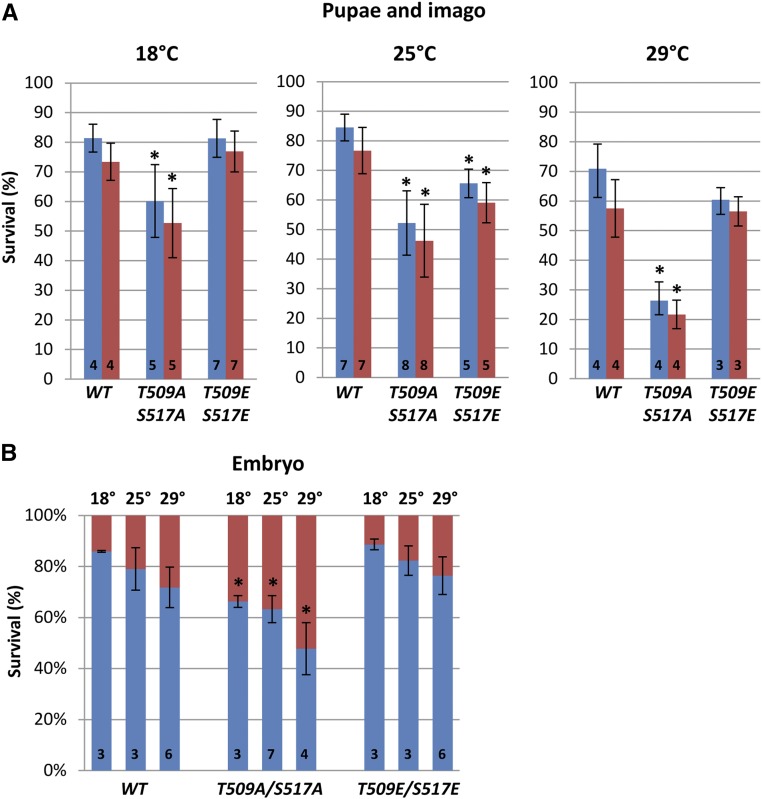
The effect of Pnut phosphorylation on *Drosophila* survival. (A) Pupae and imago survival at different temperatures. FLAG-tagged *pnut-WT*, *pnut-T509A/S517A*, or *pnut-T509E/S517E* transgenes were expressed on a *pnut^mut1^* background. The percentages of pupae formed (blue) and imagos enclosed (red) are presented. (B) Embryo viability at different temperatures. Percentages of hatched (blue) and dead embryos (red) are presented. Values at the bottom of columns represent the numbers of experiments conducted. Student’s *t*-test was used to generate *P*-values [mutants were compared to wild-type (WT)]. Asterisks indicate statistically significant difference (*P* < 0.01). Error bars represent SD.

These mutants, as well as wild-type (*pnut-WT*) transgenes, were introduced into the *Drosophila* genome (Figure S1 in File S1). First, we tested the ability of mutant transgenes to rescue the lethality associated with the *pnut^mut1^* allele at different temperatures of development (18, 25, and 29°). Both mutations were able to restore the viability of the null allele, and the obtained mutant females *pnut^mut1^/pnut^mut1^*;*pnut-T509E/S517E* as well as *pnut^mut1^/pnut^mut1^*;*pnut-T509A/S517A* were fertile. Homozygous fly stocks *pnut^mut1^*/*pnut^mut1^*;*pnut-WT*, *pnut^mut1^*/*pnut^mut1^*; *pnut-T509A/S517A*, and *pnut^mut1^*/*pnut^mut1^*; *pnut-T509E/S517E* were set up. However, the survival rates were different. Figure 3A shows pupae and imago survival. The phospho-mimetic Pnut-T509E/S517E mutant showed a statistically significant decrease in viability compared to wild- type flies at 25°, but not at other tested temperatures. In contrast, the introduction of the nonphosphorylatable Pnut-T509A/S517A mutation resulted in significantly decreased survival of flies at all tested temperatures. The difference was most prominent at 29°: 21.7 ± 4.8% of mutant flies survived to the imago stage as compared to 57.5 ± 9.7% for Pnut-WT.

Since Pnut phosphorylation was observed during the first 2 hr of development, we looked into survival rates of embryos (Figure 3B). In this experiment, we calculated the percentages of embryos that hatched to the first instar larvae. At all temperatures tested, the nonphosphorylatable Pnut mutant showed reduced embryo viability compared to wild-type. Phospho-mimetic Pnut mutant showed survival rates similar to wild-type. Therefore, we conclude that Pnut phosphorylation is important in embryogenesis as flies carrying nonphosphorylatable Pnut displayed increased lethality at the embryonic stage.

### Pnut phosphorylation is important for dynamic association of the protein at the membranes

**Figure 4:**
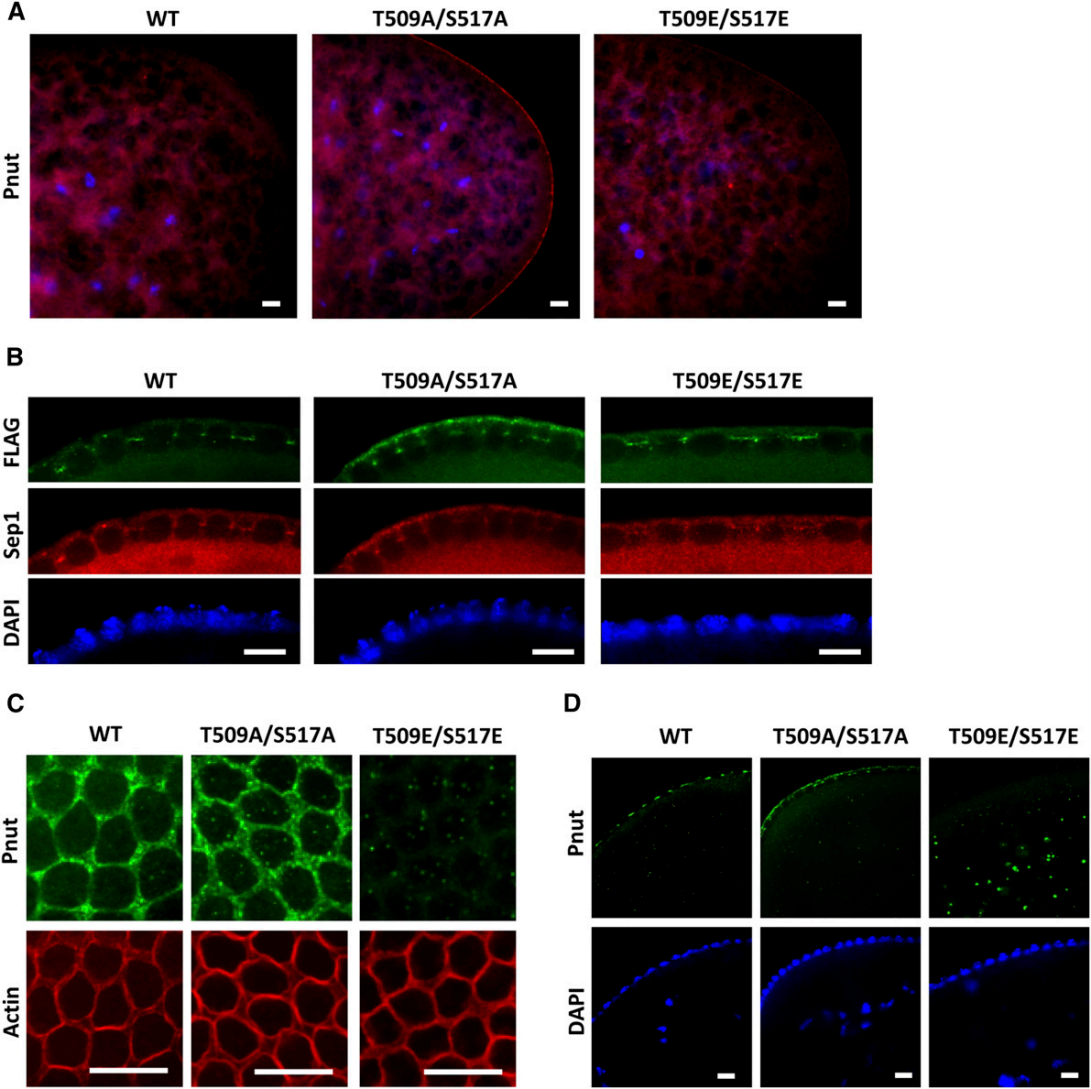
Immunofluorescent analysis of early *Drosophila* embryos. Nuclei were counterstained with DAPI (blue). (A and B) Embryos fixed using a standard technique. (A) Embryos (0–1 hr) of *pnut^mut1^* rescued with *FLAG-pnut-WT*, *FLAG-pnut-T509A/S517A*, or *FLAG-pnut-T509E/S517E* transgenes were stained for Pnut (red). (B) Embryos at the syncytial blastoderm stage (1.5–2 hr) were stained for FLAG (green) and Sep1 (red). Side views at the pseudocleavage furrows are shown. (C and D) Manual devitellinization protocol was used (see *Materials and Methods*). (C) Embryos (1.5–2 hr) were stained for Pnut (green) and actin (red). Top views at the pseudocleavage furrows are shown. (D) Embryos (1.5–2 hr) were stained for Pnut (green). Side views are shown. Bar, 10 µm. DAPI, 4’,6-diamidino-2-phenylindole; WT, wild-type.

Since the phosphorylated Pnut form is present mostly during the first 2 hr of egg development, we performed immunofluorescence analysis of the early embryos. In the early stages of *Drosophila* embryogenesis prior to nuclear migration (*i.e.*, in the first 1.5 hr of development), wild-type Pnut was found to be distributed in a diffuse manner throughout the egg, as was the case for the Pnut phospho-mimetic mutant (Figure 4A). In contrast, nonphosphorylatable Pnut protein was also found to be tightly associated with the outer embryo membrane, the only membrane that exists at this stage.

Later in egg development, during the syncytial blastoderm stage (1.5–2 hr of development), all Pnut variants were found at the pseudocleavage furrows together with Sep1 (a member of the septin complex together with Sep2 and Pnut) (Figure 4B). However, Pnut-T509A/S517A again displayed strong association with the outer membrane, contrary to wild-type and Pnut-T509E/S517E proteins. Sep1 also colocalized with Pnut-T509A/S517A at the outer membrane.

One of the steps during the preparation of *Drosophila* embryos for immunostaining is the removal of the vitelline membrane, which is impermeable to antibodies. The standard procedure includes a treatment with methanol that is known to interfere with the staining of F-actin (Muller and Sinz 2012). Septins are tightly linked to the actin cytoskeleton (Schmidt and Nichols 2004; Dolat *et al.* 2014; Kinoshita *et al.* 2002; Hagiwara *et al.* 2011) and participate in actin reorganization in early *Drosophila* embryos (Mavrakis *et al.* 2014). Therefore, to analyze the actin cytoskeleton, we performed immunostaining experiments using a method of manual devitellinization of eggs that did not include exposure to methanol (Warn and Robert-Nicoud 1990). We found that during the syncytial blastoderm stage, actin organization was normal in embryos expressing *pnut-WT*, as well as in both Pnut phosphorylation mutants (Figure 4C). However, Pnut-T509E/S517E disassociated from the membranes of pseudocleavage furrows (Figure 4, C and D) and was found in an aggregated form in the interior of the embryo (Figure 4D). The absence of phospho-mimetic Pnut-T509E/S517E in the pseudocleavage furrow during the mild fixation procedure is suggestive of a weakened association of the septin complex containing this mutant form of Pnut with the membrane.

**Figure 5:**
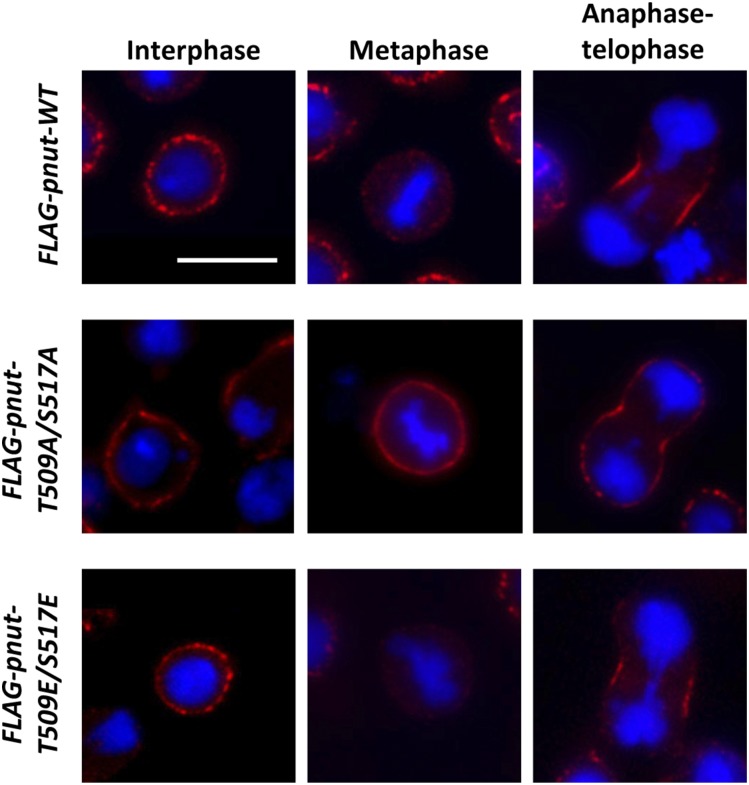
Localization of Pnut phosphorylation mutants in S2 tissue culture cells. Stable S2 cell lines carrying FLAG-tagged *pnut-WT* (upper row), *pnut-T509A/S517A* (middle row), or *pnut-T509E/S517E* (lower row) transgenes were treated with *pnut* 3′-UTR dsRNA for 5 d, fixed, and stained with antibodies against FLAG (red). Interphase, metaphase, and anaphase–telophase stages are shown. Nuclei were counterstained with DAPI (blue). Bar, 10 μm. DAPI, 4’,6-diamidino-2-phenylindole; dsRNA, double-stranded RNA; UTR, untranslated region; WT, wild-type.

To examine the membrane localization of Pnut mutants in more detail, we established stable *Drosophila *S2 cell lines expressing phospho-mimetic (T509E/S517E), nonphosphorylatable (T509A/S517A), and wild-type *pnut* transgenes. All transgenes contained the FLAG-tag sequence at the 5′-end of the *pnut* gene. Endogenous wild-type *pnut* was silenced by the treatment of cells with dsRNA complementary to the *pnut* 3′-UTR, which was absent in transgenic constructs. RNAi resulted in a near complete ablation of the endogenous protein (Figure S2 in File S1). Next, RNAi-treated cells were analyzed for FLAG-tagged Pnut protein localization in immunofluorescence experiments using the anti-FLAG antibody (Figure 5). All Pnut variants were detected at cell membranes during interphase, and at the cleavage furrow between dividing cells. However, during mitosis, 90.5% of cells expressing wild-type and 82.5% of cells expressing Pnut-T509E/S517E showed no membrane localization of FLAG-tagged Pnut (Figure 5 and Table 1). In contrast, nonphosphorylatable Pnut-T509A/S517A was found to be strongly associated with the membrane in the majority of mitotic cells (69.7%) (Figure 5 and Table 1).

**Table 1:** Pnut membrane localization in S2 cells during mitosis

	Membrane Localization (%)	No Membrane Localization*^a^* (%)	Number of Mitoses Analyzed
*PNUT-WT*	9.5	90.5	74
*PNUT-T509A/S517A*	69.6	30.4	69
*PNUT-T509E/S517E*	17.5	82.5	63

Stable S2 cell lines carrying FLAG-tagged *pnut-WT*, *pnut-T509A/S517A*, or *pnut-T509E/S517E* transgenes were treated with *pnut* 3′-untranslated region double-stranded RNA for 5 d, fixed, and stained for FLAG and 4’,6-diamidino-2-phenylindole as in Figure 5. Percentages of mitotic cells where Pnut localized at the membrane or showed diffuse staining (except for cytokinesis furrow) are shown.

a Except cytokinesis furrow.

The membrane association of the Pnut-T509A/S517A mutant regardless of cell cycle stage suggests that Pnut phosphorylation might be important for the dynamic redistribution of Pnut during the cell cycle. The phosphorylation could be necessary for the removal of the Pnut protein from the membrane during mitosis. However, we were not able to detect the phosphorylated form of Pnut in protein extracts isolated from asynchronously growing *Drosophila* S2 cells or in cells treated with colchicine, which arrests the cell population in the metaphase stage (Figure S3 in File S1). Together, immunofluorescent analysis of early *Drosophila* embryos and S2 tissue culture cells allowed us to conclude that phosphorylation of Pnut at T509 and S517 results in the decreased association of the protein with the membrane.

### The effect of Pnut phosphorylation on septin complex assembly and septin filament formation

**Figure 6:**
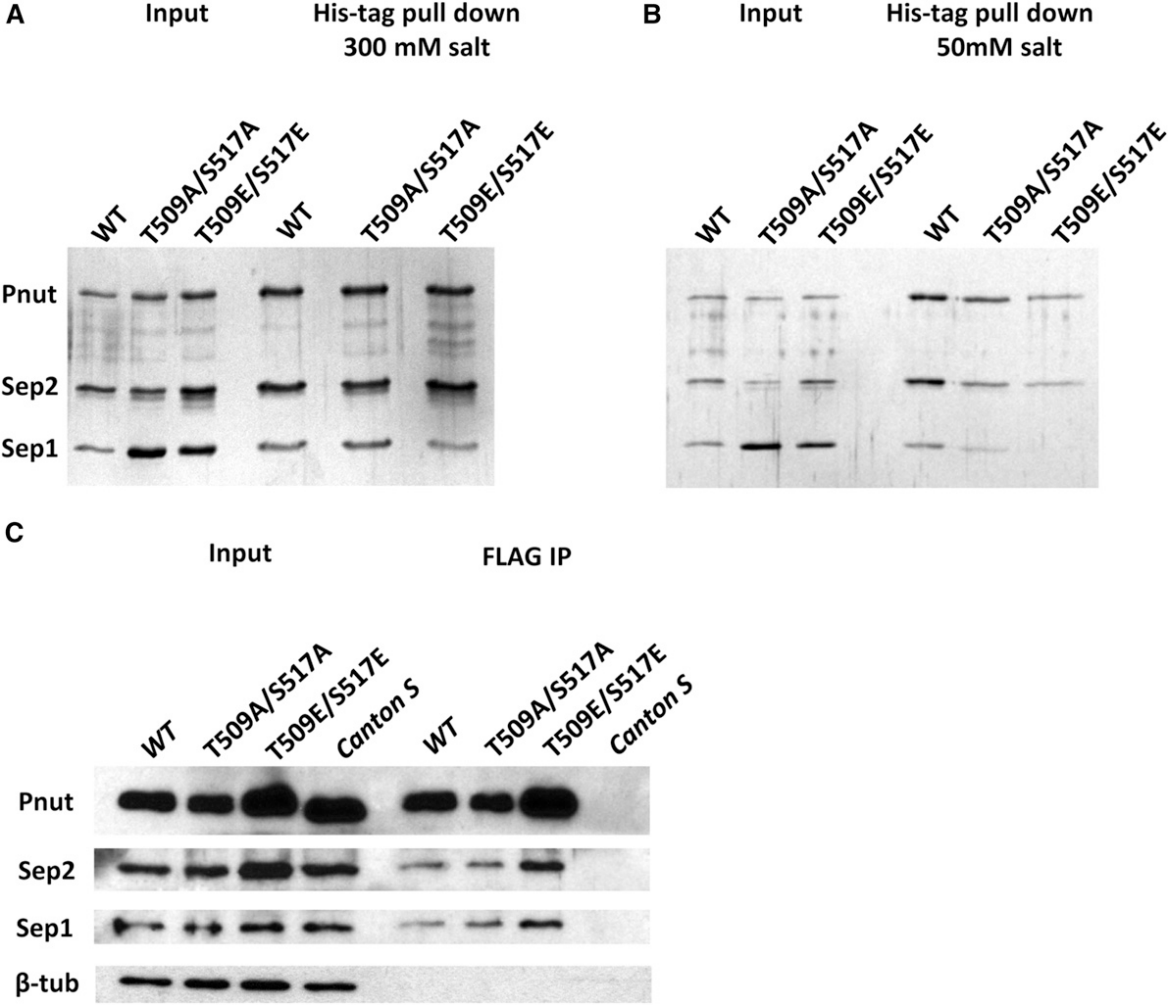
The effect of Pnut phosphorylation on septin complex assembly. (A and B) Recombinant WT or mutant septin complexes (His–Pnut)–Sep2–Sep1–Sep1–Sep2–(His–Pnut) were diluted in a buffer containing 300 mM (A) or 50 mM (B) KCl and pulled-down using Ni-NTA beads. Pulled-down materials were analyzed by silver-stained SDS-PAGE. (C) Septin complexes were immunoprecipitated from 0 to 2 hr embryonic extracts using antibody against FLAG tag. Embryos were from flies carrying FLAG-tagged *pnut-WT*, *pnut-T509A/S517A*, or *pnut-T509E/S517E* transgenes on a *pnut^mut1^* background. WT strain *Canton S* was used as a control. Immunoprecipitated material was analyzed by immunoblotting using antibodies against Pnut, Sep2, and Sep1. β-tub, β-tubulin; IP, immunoprecipitation; SDS-PAGE, sodium dodecyl sulfate-polyacrylamide gel electrophoresis; WT, wild-type.

In *Drosophila*, Pnut, Sep1, and Sep2 form a heteromeric six-subunit complex consisting of two of each septin subunits (Field *et al.* 1996; Huijbregts *et al.* 2009). To address the effect of Pnut phosphorylation on septin complex assembly, His-tagged wild-type Pnut, nonphosphorylatable Pnut-T509A/S517A, and phospho-mimetic Pnut-T509E/S517E mutant forms of protein were expressed *in vitro* together with Sep1 and Sep2 using a baculovirus expression system. Assembled complexes were purified as previously reported (Huijbregts *et al.* 2009). We then analyzed the integrity of the complex in pull-down experiments using His-tag precipitation. As shown in Figure 6A, all mutants assembled into the complex and were relatively stable in the presence of 300 mM of KCl: the amount of Sep1 (as a percentage from Pnut protein amount) was 62.3% for wild-type, 62% for Pnut-T509A/S517A, and 31% for Pnut-T509E/S517E. However, at low salt concentration (50 mM of KCl), the septin complex containing the Pnut-T509E/S517E mutant was unstable, with Sep1 protein dissociating from the rest of the complex (Figure 6B): the amount of Sep1 was only 1% for the T509E/S517E mutant (as compared to 34% for wild-type and 17% for Pnut-T509A/S517A). The result suggests that Pnut phosphorylation leads to a loose association of septin complex subunits.

**Figure 7:**
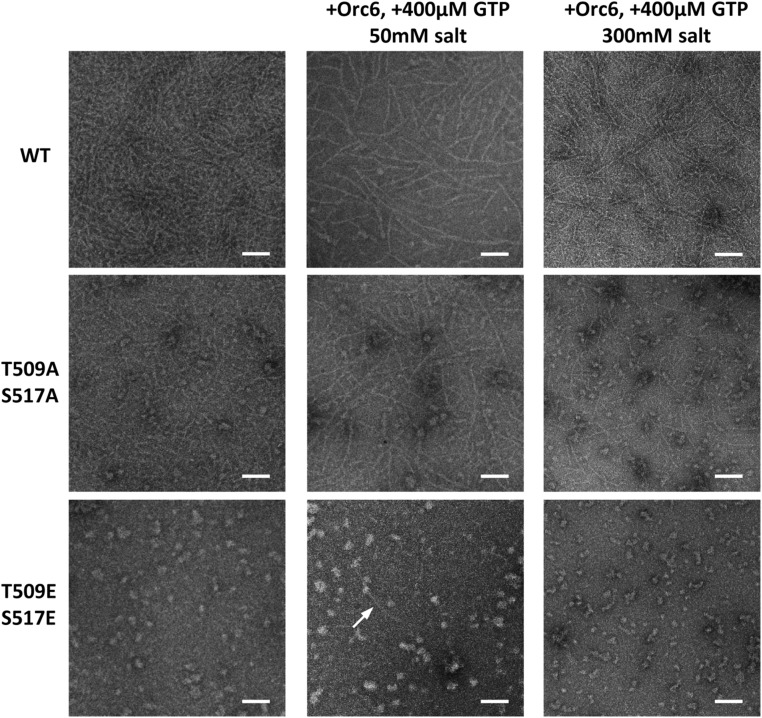
The effect of Pnut phosphorylation on septin filament formation. Recombinant wild-type (WT) or mutant septin complexes (His–Pnut)–Sep2–Sep1–Sep1–Sep2–(His–Pnut) (40 ng/µl) were incubated at different salt concentrations, with or without the addition of Orc6 (40 ng/µl) and 400 µM guanosine triphosphate (GTP) for 2 hr at 22°, and then visualized by negative-stain transmission electron microscopy. Bar, 100 nm. Arrow shows the formation of long filament in the reaction for Pnut-T509E/S517E mutant in the presence of Orc6 and GTP.

The ability to form filaments is an important characteristic of the septin complex. In our previous studies (Akhmetova *et al.* 2015), we showed that Orc6 together with GTP greatly facilitate septin filament formation *in vitro*. We reconstituted the septin complexes with wild-type or mutated Pnut proteins and analyzed septin filament formation using EM as before (Huijbregts *et al.* 2009; Akhmetova *et al.* 2015). Low salt concentrations (<75 mM KCl) are used for septin filament reconstruction since high salt (>250 mM KCl) has been shown to be detrimental for septin complex polymerization (Frazier *et al.* 1998; Bertin *et al.* 2008). At 50 mM KCl, the filament formation by the complex carrying the Pnut-T509A/S517A mutation was similar to that of wild-type flies. The complex, carrying the Pnut phospho-mimetic (Pnut-T509E/S517E) mutation had a dramatically reduced ability to polymerize (Figure 7, middle column). Instead, we observed large protein aggregates, and only few short filaments could be found when Orc6 was added to the reaction. We thought that the increase of salt concentration might improve the filament formation ability of the phospho-mimetic mutant complex (Figure 7, right column). However, no filaments were observed for the Pnut-T509E/S517E mutant complex under high salt (300 mM KCl), even though the complex was stable at this condition (Figure 6A).

To test if Pnut phosphorylation mutations have an effect on septin complex integrity *in vivo*, we examined *Drosophila* strains expressing *FLAG-pnut-WT*, *FLAG-pnut-T509A/S517A*, or *FLAG-pnut-T509E/S517E* in a *pnut^mut1^* background. Extracts were prepared from the 0–2 hr embryos and subjected to an IP experiment using an anti-FLAG antibody. The FLAG-tagged septin complexes were pulled down from extracts. As shown in Figure 6C, all septin subunits were found in pulled-down material for both Pnut phosphorylation mutants. This suggests that the septin complex might be stabilized by other factors *in vivo* that are absent in the *in vitro* experiments performed with purified septin proteins.

We also tested if Pnut phosphorylation has an effect on Orc6–Pnut interactions. The modified amino acids are located within the C-terminal domain, and we have shown before that Pnut and Orc6 interact via the C-terminal domains of both proteins (Huijbregts *et al.* 2009; Chesnokov *et al.* 2003). We used antibodies raised against the Orc6 protein to precipitate Pnut from *Drosophila* embryo extracts and found that both mutant forms of Pnut were able to interact with Orc6 in the IP experiments (Figure S4 in File S1).

## Discussion

Post-translational modifications of septins are described in many different organisms from yeast to mammals. The most common modification is a phosphorylation, which has been shown to have an effect on septin dynamics (Tang and Reed 2002; Versele and Thorner 2004; Garcia *et al.* 2011; Sinha *et al.* 2007; Meseroll *et al.* 2012, 2013; Amin *et al.* 2008; Koch *et al.* 2015). In this work, we discovered that *Drosophila* septin Pnut is phosphorylated during early embryogenesis. Mass spectrometry has identified that the phosphorylation occurs at the extreme C-terminus of the protein on amino acids T509 and S517. One of these phosphorylation sites (S517) was also found during mass spectrometry-based phosphoproteome analysis of *Drosophila* embryos; however, no functional studies were performed (Zhai *et al.* 2008).

Members of the septin family vary greatly in terms of the sequence and length of their N- and C-termini (Hall and Russell 2004). In the crystal structure of the human SEPT2/6/7 complex (Sirajuddin *et al.* 2007), the extreme N and C termini are disordered, and consequently the roles of these regions are not completely understood. It is possible that either or both may be involved in septin–septin interactions (Sala *et al.* 2016). In our earlier studies (Huijbregts *et al.* 2009), we showed that deletions of the C-terminal domain of Pnut resulted in its inability to interact with other septins and form a functional *Drosophila* septin complex (Pnut–Sep2–Sep1). Thus, it is possible that modifications within the C-terminus of Pnut may have an effect on septin complex assembly and function.

In order to study the effect of Pnut phosphorylation in early *Drosophila* development, we have created a new *pnut* null mutant. The advantage of our *pnut^mut1^* is that homozygous adult females rescued by the wild-type *pnut* transgene are fertile, which allows the analysis of various *pnut* mutations during embryogenesis.

We used transgenic *Drosophila* strains to examine the role of Pnut phosphorylation *in vivo* in live animals. The substitution of the endogenous Pnut with the nonphosphorylatable mutant form resulted in increased lethality at the embryonic stage at 18, 25, or 29°. On the other hand, the phospho-mimetic mutant showed no significant decrease in hatched embryos compared to wild-type at all analyzed temperatures. These findings indicate that phosphorylation is important in early embryogenesis.

Our immunolocalization experiments performed in early embryos as well as in tissue culture cells demonstrated that nonphosphorylatable Pnut protein was more often found at the membrane than the wild-type protein. These results suggest an increased stability of membrane-associated septin structures formed by this mutant form of Pnut. Association with the cell membrane of the phospho-mimetic mutant was similar to that of wild-type Pnut. However, using a different method of embryo fixation, we also revealed that this mutant form of Pnut more loosely associated with membranes compared to the other Pnut proteins tested.

Our results are similar to those in the filamentous fungus *A. gossypii*, (Meseroll *et al.* 2012, 2013), which revealed that septin Shs1 can be phosphorylated at multiple sites, including two phosphorylation sites at the very end of the C-terminus, just outside the coiled-coil domain, as is the case for *Drosophila* Pnut. The nonphosphorylatable Shs1 mutant was more concentrated at the septin rings at the membrane and displayed reduced septin dynamics (Meseroll *et al.* 2012), whereas the dynamics of the phospho-mimetic mutant protein were significantly increased relative to those of the wild-type protein (Meseroll *et al.* 2013). Another example is the phosphorylation of the extreme C-terminus of *S. cerevisiae* septin Cdc3. This domain is required for the disassembly of the membrane-associated septin ring. The nonphosphorylatable mutant form of Cdc3 (serines substituted with alanines) exhibited increased stability of the septin ring (Tang and Reed 2002).

To investigate the molecular mechanisms standing behind the phenotypes observed with *Drosophila* Pnut mutant proteins, we reconstituted the recombinant septin complexes carrying phosphorylation mutations using a baculovirus expression system. We found that the septin complex containing the phospho-mimetic Pnut mutant form was unstable at low (50 mM) salt concentration. As a consequence, this complex had a tendency to form aggregates and showed a dramatically reduced ability to polymerize and form long septin filaments *in vitro*. Similarly, in mice testes, increased phosphorylation of SEPT4 interfered with the ability of this particular septin to form high-molecular weight complexes and, as a result, compromised septin membrane barrier function (Koch *et al.* 2015).

Together, our data suggest that Pnut phosphorylation may facilitate septin complex and higher septin structure disassembly during specific stages in *Drosophila* embryo development and/or during the cell cycle. Interestingly, in yeast and *A. gossypii*, cytoplasmic septins are thought to exist primarily as single complexes. Complexes from the cytosol come together at the cell membrane and form long filaments by a process called annealing (Bridges *et al.* 2014). If *Drosophila* septins employ a similar mechanism, the phosphorylation of Pnut could be useful to inhibit filament growth and facilitate the removal of septins from the membrane. Such disassembly may be important during syncytial divisions. After the migration of nuclei to the embryo cortex, division cycles 10–13 are very fast (∼10 min each), and require quick and synchronous pseudocleavage furrow ingression and retraction between adjacent dividing nuclei. When assembled on the cell membrane, septin filaments are shown to influence the shape of animal cells and provide rigidity to the cortex (Gilden and Krummel 2010). Therefore, phosphorylation of T509 and S517 in the Pnut protein might help embryos to proceed through embryogenesis by supporting membrane plasticity during the first hours of development.

Pnut phosphorylation is detected during the first 2 hr of embryo development and disappears after cellularization. A study from the Peifer laboratory revealed that Pnut-deficient embryos were able to complete early stages of syncytial development but failed at the later stages (Adam *et al.* 2000). The subtle defects during cellularization were followed by obvious morphological defects at gastrulation. We hypothesize that Pnut function may be dispensable at the syncytial stage and may be even harmful to the embryo. Accordingly, the observed phosphorylation of Pnut in our studies might serve to specifically deactivate some of the Pnut functions resulting in septin complex disassembly and defects in septin polymerization. This regulation may be part of a mechanism to prevent premature cellularization. Importantly, flies carrying nonphosphorylatable Pnut mutations displayed significantly lower rates of embryo survival, suggesting that its inability to be phosphorylated results in the accumulation of errors during embryogenesis and overall decreased survival rates.

Also, the dissociation of the septin complex due to phosphorylation may give way to the incorporation of other septin subunits. All five *Drosophila* septins—Pnut, Sep1, Sep2, Sep4, and Sep5—are expressed during early embryogenesis (Graveley *et al.* 2011). Septins are shown to replace each other in complexes (Sandrock *et al.* 2011; Sellin *et al.* 2011, 2012). *Drosophila* Sep4’s sequence resembles that of Sep1, *Sep5* represents a retrogene copy of *Sep2*, and the coding proteins share a very high level of homology (Betran *et al.* 2002). Therefore, Sep4 and/or Sep5 might substitute for Sep1 and/or Sep2, respectively, in the complex, destabilized by Pnut phosphorylation. Colocalization data suggests that the alternative complex containing Pnut, Sep4, and Sep5 might exist during early syncytial division cycles in *Drosophila* embryos (Su *et al.* 2013).

Despite its inability to form filaments *in vitro*, the phospho-mimetic mutant protein was similar to that of wild-type Pnut in the survival experiments. In addition, IP experiments using extracts isolated from embryos expressing Pnut-T509E/S517E protein suggest that the functional septin complex can be assembled *in vivo*. It is possible that the *in vitro* system lacks some key components that might help septin complexes to assemble into filaments *in vivo*. One of these factors could be cell membrane itself. In yeast, the interaction with the membrane (particularly with phosphatidylinositol) promotes septin filament formation even in the case of mutations that prevent polymerization in solution (Bertin *et al.* 2010). Other possible factors could be interacting partners. In our previous works, we found that Orc6 facilitates filament formation of the septin complex in *Drosophila*. Orc6 molecules bind Pnut subunits of two adjacent septin hexamers (Pnut–Sep2–Sep1–Sep1–Sep2–Pnut) and bring them together to form filaments *in vitro* (Huijbregts *et al.* 2009; Akhmetova *et al.* 2015). *In vivo* IP from early embryos revealed that the ability of the phospho-mimetic mutant to interact with Orc6 was not compromised. Therefore, Orc6 along with a membrane might promote septin filament formation *in vivo*.

The following question arises: does Pnut phosphorylation occur routinely during mitosis and, therefore, can it help septin structures disassembly during every cell cycle? In the early *Drosophila* embryo, nuclei divide synchronously in the syncytium, and the cell cycle consists of only S and M phases without detectable gap phases (Orr-Weaver 1994; Vidwans and Su 2001). Therefore, the percentage of mitotic cells may be high enough to allow the easy detection of Pnut phosphorylation if we assume that Pnut phosphorylation is cell cycle-specific. We were not able to detect Pnut phosphorylation in other *Drosophila* tissues or in *Drosophila* culture cells, even when cells were arrested at mitosis by colchicine treatment. However, labeling *Drosophila* culture cells with radioactive orthophosphate revealed the presence of phosphorylated Pnut in these cells (Figure S5 in File S1). Our future studies with synchronized cell cultures and scaled-up mass spectrometry analysis of isolated phosphorylated proteins will help to reveal whether Pnut phosphorylation in cell lines occurs at the same sites that were observed in early embryos. The possibility exists that the secondary phosphorylated sites might appear even in phospho-mimetic and/or nonphosphorylatable Pnut mutants. In yeast and filamentous fungi, septin phosphorylation has been detected at multiple sites (Hernandez-Rodriguez and Momany 2012). Moreover, there are many examples of compensatory phosphorylation events resulting in other sites being phosphorylated when the preferred amino acids are mutated (Bauer *et al.* 2003; Zheng *et al.* 2010; Schwarz *et al.* 1996; Gao *et al.* 2013).

Overall, the data presented in this study support the idea that septin phosphorylation might affect septin organization into higher-order structures and their dynamics and, as a consequence, the viability of multicellular organisms. It also demonstrates that *Drosophila* provides a useful model system for our greater understanding of the diverse effects of septin modifications.

## Supplementary Material

Supplemental material is available online at www.g3journal.org/lookup/suppl/doi:10.1534/g3.117.300186/-/DC1.
