# Supplementary material for: Phosphorylation of Pnut in the Early Stages of Drosophila Embryo Development Affects Association of the Septin Complex with the Membrane and Is Important for Viability
Source: G3 (Bethesda). 2017 Oct 17;8(1):27–38. doi: 10.1534/g3.117.300186 (PMC5765355; doi:10.1534/g3.117.300186)
Supplement: Supplementary file 1 [file 27FileS1.pdf]

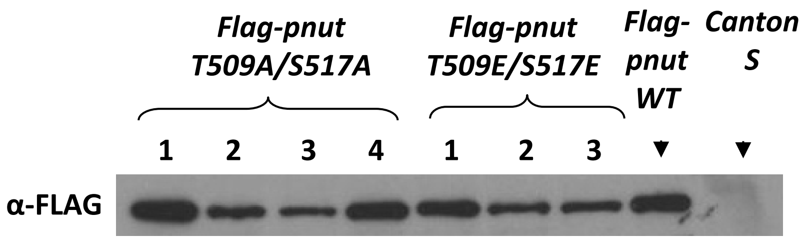

Figure S1. Confirmation of *pnut* transgenes expression.

Ovary extracts from transgenic flies containing FLAG tagged *pnut-WT, pnut-T509A/S517A* or *FLAG-pnut-T509E/S517E* were subjected to immunoblotting using anti-FLAG antibody. The presence of the FLAG-tagged product was confirmed for all strains analyzed (four for T509A/S517A, three for T509E/S517E and one for WT).

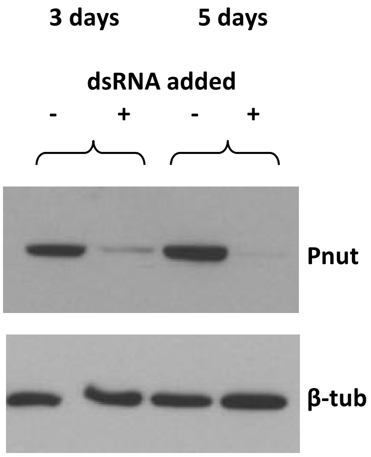

Figure S2. ***Pnut*** **RNAi in** ***Drosophila S2*** **cells using dsRNA against** ***pnut*** **3'-UTR.** 1 x 10^6 *S2* cells were incubated with 15 µg of dsRNA complementary to *pnut* 3'-UTR. No dsRNA were added to the control cells. After 3 or 5 days, RNAi efficiency was tested by immunoblotting with antibody against Pnut. β-tubulin serves as a loading control.

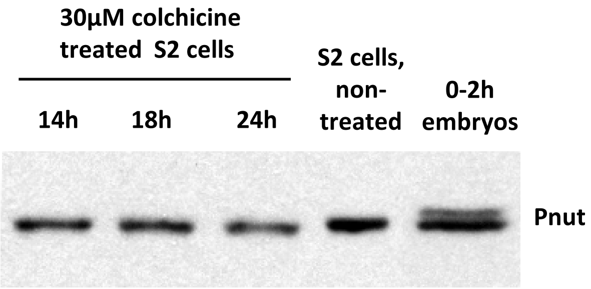

Figure S3. Pnut phosphorylation is not detected in *Drosophila S2* cells.
S2 tissue culture cells were treated with 30 µM colchicine to enrich mitotic stage. After 14, 18 or 24 hours, the extracts were prepared and analyzed by Western blot using antibodies against Pnut. 0-2 hours embryonic extract was loaded as a control for Pnut T509/S517 phosphorylated form.

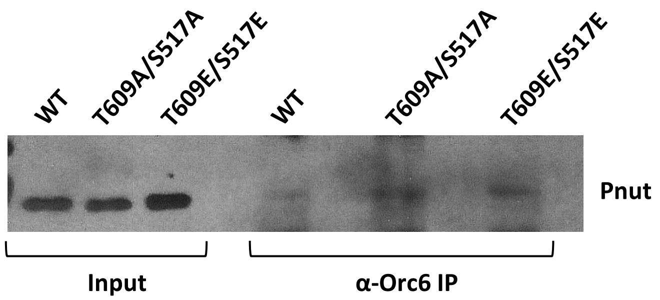

Figure S4. Mutations in Pnut phosphorylation sites do not affect interaction with Orc6.

100 embryos of 0-12 hour age were collected from flies of genotype *pnut*^{mut1}/*pnut*^{mut1};*FLAG-pnut* wild type or with mutated phosphorylation sites. Embryos were lysed in an IP buffer supplemented with 500 µM GTP. Orc6 antibodies were used for immunoprecipitation. Immunoprecipitated material was analyzed by SDS-PAGE followed by immunoblotting using antibody against Pnut.

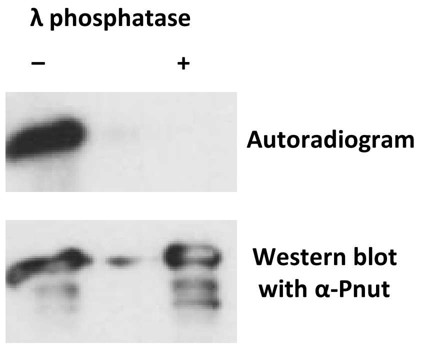

Figure S5. Incorporation of radioactive phosphate by Pnut in *Drosophila S2* cells.

S2 cells were labeled with ^{32}P as described previously (Remus *et al.*, 2005), except no TBB was added. Pnut was immunoprecipitated from labeled whole-cell extract using anti-Pnut antibody. Half of the sample was treated with phage λ protein phosphatase (NEB). Samples were fractionated by SDS-PAGE and transferred to PVDF membrane. After exposure of the membrane to autoradiography film, the membrane was probed with antibodies against Pnut.
